# Preliminary Benefits of In-Home Virtual Reality for Chronic Pain in Sickle Cell Disease: Pilot Randomized Trial

**DOI:** 10.3390/biomedicines14061334

**Published:** 2026-06-12

**Authors:** Nadine Matthie, Melinda Higgins, Coretta Jenerette

**Affiliations:** 1 Nell Hodgson Woodruff School of Nursing, Emory University, 1520 Clifton Road NE, Atlanta, GA 30322, USA; melinda.higgins@emory.edu; 2 Community Health Systems, School of Nursing, University of California San Francisco, 490 Illinois Street, Floor 9, Box 0608, San Francisco, CA 94143, USA; coretta.jenerette@ucsf.edu

**Keywords:** virtual reality, sickle cell disease, chronic pain, home-based pain management, adults

## Abstract

**Background/Objectives**: Non-pharmacological approaches are urgently required for managing chronic pain in sickle cell disease (SCD). This multiple-method, exploratory pilot randomized trial was conducted to evaluate preliminary benefits of an in-home virtual reality (VR) intervention (EaseVRx), compared to content-matched audio attention control, for chronic pain reduction and changes to chronic pain-related outcomes in adults with SCD. **Methods**: Participants were randomized to VR (19) or audio (25), with 2–16 min daily modules for eight weeks, a daily pain diary survey, VR surveys, assessments of chronic pain grade and chronic pain correlates every 4 weeks for 3 months, and a qualitative interview. Analyses were conducted using quantitative and qualitative methods. Clinical Trial Registration: ClinicalTrials.gov, NCT04906707, 27 May 2021. **Results**: Average intervention use was 19.1 and 12.1 min per day in VR and audio, respectively. Preliminary benefits of VR included decreased pain (*p* = 0.002), improved pain coping (pain catastrophizing [*p* = 0.005] and chronic pain acceptance [*p* = 0.002]), and improved sleep (*p* = 0.009). Additionally, symptoms associated with cybersickness were significantly lower (*p* = 0.012) after VR use than before VR use. Among interviewees, the interventions were acceptable and helpful in managing pain, although there were some barriers and challenges to use. **Conclusions**: Study findings suggest that in-home VR may be a self-management resource for adults with SCD and warrants additional research.

## 1. Introduction

Sickle cell disease (SCD) is an inherited group of red blood cell disorders that is caused by a mutation in the hemoglobin-Beta gene, which results in sickling of the red blood cells [1,2,3]. SCD is associated with long-term complications (including pain and organ damage), frequent hospitalizations, and high healthcare utilization and indirect costs [4]. As of 2021, at least 8 million people were living with SCD worldwide, with individuals of African, Mediterranean, Middle Eastern, and Indian descent being most affected [3]. Healthcare systems, resources, and sociocultural factors vary by world regions, yet there is a consistent global pattern of need in relation to SCD. Cross-regional issues include complex clinical needs (e.g., chronic pain), access challenges (e.g., to resources and multimodal pain management), stigma and structural inequity (e.g., associated with race and marginalization or poverty), and insufficient investment relative to the burden of SCD [5,6].

In the United States (U.S.), SCD affects more than 100,000 people and more than 90% are non-Hispanic Black or African American [7]. These adults with SCD are an underserved population with unmet needs for chronic pain management. Chronic, non-vaso-occlusive pain in SCD is defined as ongoing pain that was present, in one or more locations, on most days for more than six months [8]. It is attributed to factors including avascular necrosis of joints, bone infarction, leg ulcers, osteomyelitis, and central and peripheral sensitization [8,9,10,11,12]. Of adults with SCD, 84% report experiencing chronic non-vaso-occlusive pain [13]. The pain occurs in at least two body sites (61.1%) and the back is the most common pain site (61.1%) [14]. Chronic pain in SCD is associated with negative coping behaviors [15,16], functional disability [13], poor quality of life (QoL) [17], and high healthcare utilization [17,18]. It also contributes to sleep disturbances [19,20,21], anxiety and depression [20,21], which in turn, exacerbate the pain burden [19,22]. Taken together, these factors contribute to lost productivity, morbidity, and mortality [13,23,24,25]. Opioids are typically used to manage chronic pain, but their access may be limited because of the opioid epidemic; they are ineffective, and they are associated with greater pain (due to hyperalgesia or sensitization), poorer functional outcomes, and higher healthcare utilization [26,27,28,29,30,31]. Non-opioid analgesics (e.g., nonsteroidal anti-inflammatory drugs), adjuvant analgesics (e.g., gabapentin), and multimodal pharmacological approaches (e.g., non-opioids along with adjuvants) have been evaluated in adults with SCD. Major goals of using these other pharmacological approaches are to reduce reliance on opioids and mitigate opioid-induced hyperalgesia [32]. However, the evidence base is limited [32,33,34] and the presence of neuropathic pain, for which adjuvants would be employed, is underrecognized [35]. Therefore, non-pharmacological approaches are urgently needed to manage chronic pain in SCD.

Behavioral pain management strategies are being increasingly used for chronic pain as an alternative to opioids, but adults with SCD encounter challenges in accessing these resources [36]. Although the type and scope of challenges in accessing behavioral pain management strategies varies by world region, given differences in healthcare systems and resources, shared challenges across regions include limited availability of behavioral interventions/services, lack of integrated care models, socioeconomic inequities, and stigma and discrimination [37,38,39]. In the U.S., factors such as discrimination and negative effects of social determinants of health influence access to and utilization of mind–body interventions [13,14,26,27,40]. Consequently, home-based self-care is a mainstay of chronic pain management, especially for 18–40-year-olds with SCD who experience a high incidence of chronic pain and chronic pain disability [13]. However, adults with SCD reported a need for better strategies and new resources to support their management of pain at home [13,14]. In-home virtual reality may meet this need.

Virtual reality (VR) incorporates computer graphics, sensory input devices, visual displays, sounds, and other sensations to create an immersive, virtual environment [41]. VR has improved pain, pain-related outcomes, and self-efficacy in chronic pain conditions [41,42] through pain distraction and engaging stimuli [43], and cortical re-patterning that produces analgesia [43,44]. Although VR use can cause cybersickness or digital motion sickness [45], most users report being satisfied with VR as an acceptable intervention for chronic pain [41]. Many VR interventions used for pain management focus on cognitive and/or attentional distraction [41]. While this mechanism of action can be helpful for acute pain in SCD, a different methodology is necessary for chronic pain in SCD. According to the biopsychosocial–spiritual (BPS–spiritual) model of chronic pain in SCD, chronic pain has biological, psychological, social, and spiritual dimensions [46]. Biological factors include SCD genotype, age, and sex [46]. Psychological factors include catastrophizing, emotional distress, and coping strategies [46]. Social factors include socioeconomic status, activities of daily living (ADLs), and social/family support [46]. Spiritual factors include religious or spiritual beliefs, religious coping styles, spiritual distress, and forgiveness and inner peace [46,47,48]. Therefore, a multidimensional approach is required for addressing chronic pain in SCD. AppliedVR’s (AVR, Inc.; Van Nuys, CA, USA) EaseVRx may be used to implement this type of approach.

EaseVRx is a commercially available, 8-week, skill-based, immersive VR program developed for in-home, adjunctive treatment of chronic lower back pain in adults, which was designed to minimize triggers of cybersickness [49]. Although EaseVRx was developed for people who experience chronic lower back pain, the content addresses chronic pain more broadly, with program modules that incorporate the following: (1) pain edu-cation (to develop pain coping skills); (2) pain psychology (based on cognitive behavioral therapy for pain, to encourage adoption of active problem-solving to cope with challenges associated with pain); (3) biofeedback training (to regulate the sympathetic nervous system and reduce symptoms of hyper-arousal of the nervous system by controlling breathing); (4) relaxation using mindfulness-based stress reduction (to encourage awareness of the mind and the body’s role in pain); and (5) distraction through interactive games [49]. Mechanisms of action include limiting pain signal processing by stimulating the visual cortex and other senses [43], activating descending inhibitory pathways and inhibiting spinal transmission of peripheral afferent pain signals [50,51], shortening pain episode perception via effects on prefrontal time perception [52], and providing an immersive environment for developing and practicing specific skills [53]. In chronic lower back pain, fibromyalgia, rheumatoid arthritis, and lupus, EaseVRx safely reduced pain intensity, pain interference, and psychological correlates of pain [41,42]. EaseVRx is applicable to chronic pain in SCD given reports among adults with SCD of the back as the most common body site for experiencing chronic non-vaso-occlusive pain [14], neuropathic and nociceptive issues that contribute to central sensitization like in chronic lower back pain [54], and commonalities (e.g., chronic inflammation and musculoskeletal pain) with chronic pain conditions like lupus for which VR has been beneficial [41,42]. EaseVRx may influence chronic pain and chronic pain correlates among adults with SCD by targeting two of the four constructs in the BPS–spiritual model of chronic pain in SCD: psychological and social. Additionally, chronic pain in SCD may respond differently to VR because chronic pain onset is associated with a process of re-wiring the brain, which results in a lower pain perception threshold [55].

Initial evaluations of EaseVRx among two small and different samples of adults with SCD suggest that VR may be a useful and feasible strategy for non-pharmacological management of their chronic pain [56,57]. In a qualitative study (*n* = 9), adults with SCD who were exposed to EaseVRx reported that their pain experience was positively influenced by the VR program, and most (88.9%, *n* = 8) viewed it as a useful tool for supplementing their current pain management strategies [56]. In a subsequent feasibility study (*n* = 19), participant enrollment and intervention use were feasible [57]. However, follow-up questionnaire response rates were <50%. There were also reports of slight symptoms associated with cybersickness (primarily general discomfort, fatigue, and headache) among seven of ten VR users, but there was no VR-associated nausea and symptoms associated with cybersickness did not appear to hinder program use [57].

For adults with SCD, in-home VR therapies may be more efficacious and result in fewer complications than opioid therapy. Findings of initial evaluations of VR in adults with SCD substantiated the need for additional research to examine that potential, while further assessing questionnaire response and symptoms associated with cybersickness. No known randomized trials have investigated the benefits of in-home VR on chronic pain in adults with SCD. Therefore, this exploratory pilot randomized trial, which incorporated qualitative methods, evaluated the preliminary benefits (for chronic pain reduction and changes to chronic pain correlates) of using EaseVRx, versus audio attention control, as an in-home VR intervention for chronic pain self-management among adults with SCD. It was hypothesized that preliminary benefits would include decreased pain; improved pain coping, sleep, and functional ADLs; decreased disability; and enhanced QoL. The trial was pre-registered at ClinicalTrials.gov (number NCT04906707; registered 27 May 2021). Study reporting was guided by the Consolidated Standards of Reporting Trials (CONSORT) Statement [58] and the Consolidated Criteria for Reporting Qualitative Research (COREQ) [59].

## 2. Materials and Methods

### 2.1. Study Design, Sample, and Recruitment

After obtaining Institutional Review Board approval (IRB00002004) from the university, this exploratory, multi-method, two-group, parallel, pilot randomized trial engaged adults (ages 18–50) with SCD in the U.S. Preliminary power calculations, performed using International Business Machines Corporation’s (IBM; Armonk, NY, USA) Statistical Package for the Social Sciences (SPSS) (version 27) [60], indicated that a sample size of 60 would enable detection of a moderate (Cohen’s *d* = 0.5) to large (*d* = 0.8) effect size with adequate power (80%) at a 5% significance level. To obtain a sample of 60, enrollment of 72 participants was projected to account for a potential 20% dropout or non-completion rate.

Participants were enrolled, in person or remotely, between October 2021 and December 2023 using the inclusion and exclusion criteria listed in Table 1. The exclusion criteria reflect conditions that may have posed safety risks or interfered with successful engagement and completion of study activities. Individuals were recruited through SCD clinic visits, study flyers, social media in collaboration with a SCD foundation, snowball recruitment, and direct contact with adults with SCD in the Southeastern U.S. who participated in a previous descriptive study of chronic pain [13]. For participants who were enrolled in person, SCD status was confirmed through records of the SCD clinic or SCD foundation. For participants who were enrolled remotely, SCD status was self-reported. However, adults recruited from the prior study had their diagnosis previously verified through electronic health records (EHRs), providing additional confirmation for a portion of this study’s sample. Because recruitment occurred through and the study was promoted by SCD clinics, a SCD foundation, and adults with SCD (whose SCD status was confirmed via the EHR for a previous study), self-report of SCD type during screening was considered acceptable. Screening also included questions about usual source of SCD care, sickle cell type, pain score and lab values (including hemoglobin) at most recent clinic visit, past medical history (diagnoses, medical conditions, and symptoms experienced), past surgical history, current medications (name, dose, and frequency at which it must be administered), number of pain crises experienced in the past year, number of hospital visits (emergency department visits versus hospital admissions) with pain as the presenting/admitting problem in the past year, and number of clinic visits in the past year. Individuals who were unable to answer those questions were screened out.

### 2.2. Study Randomization and Procedures

**Figure 1:**
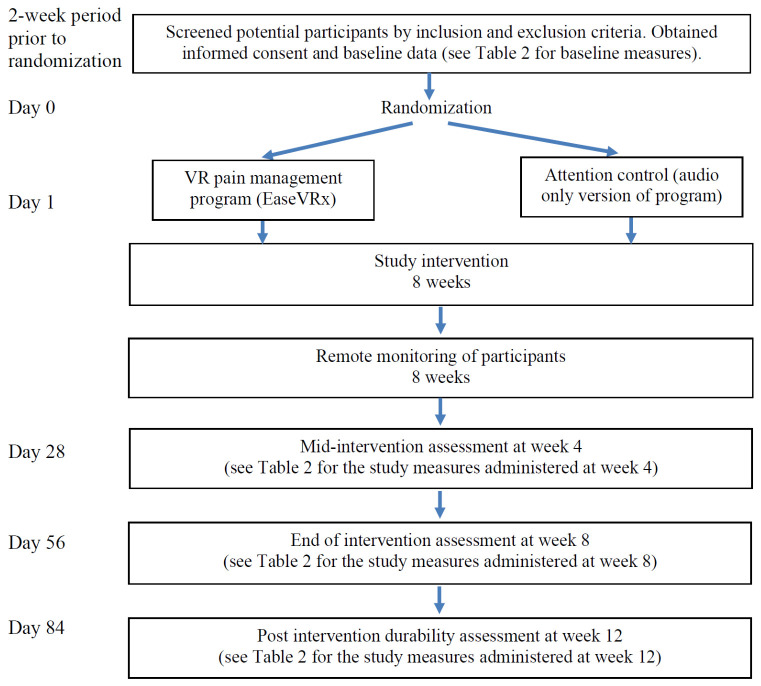
Study timeline.

The timeline of the trial is depicted in Figure 1. Informed consent was obtained, in person or remotely, before administration of electronic baseline questionnaires via study iPad in person or remotely via the web. After baseline data collection, 44 individuals were randomized 1:1 to one of the two study interventions, VR (AVR’s EaseVRx; *n* = 19) or audio (AVR’s audio-only version of EaseVRx; *n* = 25). Using version 9.4 of Statistical Analysis System (SAS; SAS Institute Inc., Cary, NC, USA) software [61], the study statistician employed a stratified, randomized block design to generate the group assignment sequence. The strata were created using biological sex (male and female) and each stratum consisted of a block size of 6. To conceal the random allocation sequence until intervention assignment and implement the sequence, a sequentially numbered, sealed envelope system was utilized by the study staff member who was enrolling participants. Given the type of interventions being used in the study and associated data collection, it was not possible to blind participants to intervention assignment. As the analyst, the study statistician remained blinded to group assignments until after the data were collected.

After group assignment, study participants were followed prospectively for 8 weeks during intervention use at home. Using a web link, they were also asked to remotely complete these electronic post-baseline surveys: daily pain diary surveys (until the end of week 8), and repeated surveys administered at mid-treatment (week 4), post-treatment (week 8), and follow-up (week 12). Information about the specific personal electronic devices (such as a smartphone, tablet, laptop, or desktop computer) used by individual participants to remotely complete the electronic assessments was not collected. Participants received $200 in gift cards as incentives: $50 after baseline and $50 each after weeks 4, 8, and 12.

### 2.3. Study Interventions

#### 2.3.1. Virtual Reality (VR)

EaseVRx consists of 56 daily modules, ranging from 2 to 16 min (average 6 min), presented via immersive and interactive three-dimensional visual environments [49]. The environments presented within the virtual setting include natural/scenic worlds (e.g., oceans), gamified environments (e.g., targeting attention training and executive function), and distraction experiences (e.g., swimming with dolphins). The program modules—core types being pain education, cognitive/behavioral training, mindfulness and awareness, and behavior change—enable users to perform guided therapeutic exercises focused on breathing and relaxation, mindfulness, body awareness, attention shifting, and cognitive and executive function. An example of a VR module is Dynamic Breathing, wherein users are instructed to perform diaphragmatic breathing. As users practice this strategy, they are asked to pace their breathing by “breathing life into a tree” while the on-screen tree is illuminated in conjunction with the exhalation.

Participants randomized to VR received a wireless VR headset (Pico G2 4K headset (Pico Technology Co., Ltd.; Qingdao, China) with AVR breathing amplifier (AVR, Inc.; Van Nuys, CA, USA)), pre-loaded with EaseVRx, either in person or via shipment. Study staff provided a brief device tutorial, either in person or remotely (via Zoom [Zoom Video Communications, Inc.; San Jose, CA, USA; versions 5–6] or telephone, based on the participant’s preference), using AVR instructional manuals and the study protocol. The tutorial involved guiding participants through initial device set up and use. Participants also received AVR’s technical support contact information. Each week, VR group participants were asked to complete 7 modules in the prescribed sequence pre-determined by the program. They could repeat modules if desired and they also had access to the EaseVRx library that contains additional content such as relaxation videos and interactive games. To decrease the risk of cybersickness and enhance safety, participants were advised to use the device while seated, limit use to three times during 24 h for no more than 30 min consecutively, and to remove the device if there was any discomfort.

#### 2.3.2. Audio

Participants randomized to audio received access to AVR’s audio-only version of EaseVRx that consisted of 56 daily modules (ranging from 2 to 16 min, average 6 min). The audio modules, which contained the same program content as the VR intervention, were delivered through voice-guided audio sessions (that included narration, guided imagery, and auditory coaching) rather than immersive visual environments [42]. An example of an audio module is Meditation: Mindful Breathing. In this module, users are guided to focus on breath awareness and relaxation while completing deep breathing exercises to the backdrop of sounds of ocean waves and new-age music. The audio program, which was well received by participants in a prior study of adults with SCD [57], was selected as an attention control to facilitate a preliminary evaluation of the benefits of VR immersion in improving chronic pain and chronic pain correlates. Additionally, use of this attention control enabled an exploration of the differential benefits of a multi-sensory versus auditory-only intervention on chronic pain and chronic pain correlates as well as a comparison of engagement with the two interventions.

Participants in the audio group received an electronic link to the audio modules on SoundCloud (Berlin, Germany; versions 2022–2024), an online music and audio distribution platform, which they could stream via any electronic device (such as a smartphone, tablet, laptop, or desktop computer). Study staff provided instructions regarding use of SoundCloud. Each week, audio group participants were asked to complete 7 modules in the prescribed sequence pre-determined by the program, and they could repeat modules if desired. Information about the specific personal electronic devices used by individual participants to access the audio program via SoundCloud was not collected.

### 2.4. Study Outcomes and Measures

Study outcomes were chronic pain, chronic pain correlates, and symptoms associated with cybersickness. Chronic pain correlates were chronic pain disability, pain catastrophizing, chronic pain acceptance, chronic pain self-efficacy, social support, health literacy, executive function, anxiety, depression, sleep impact, functional ADLs (pain impact), and QoL. These correlates were selected based on either their inclusion in core outcome domains for chronic pain [62,63], findings of previous chronic pain and VR investigations among adults with SCD [13,14,57], their importance to adults with SCD with respect to QoL [13,14], or their established reliable and valid measures [57]. Additionally, study measures assessed demographics, baseline SCD severity and cybersickness risk, symptoms associated with cybersickness, daily pain and self-management, and intervention use. Because the study was an exploratory pilot trial, primary outcomes (or composite outcome measures) were not defined a priori. To evaluate study outcomes, several self-administered, patient-reported outcome measures were utilized throughout the study as surveys. The description (including validity), administration, and reliability of the study measures are provided in Table 2.

Intervention use (set at 2–16 min per day) was measured with VR device usage statistics (minutes of use and use of modules versus library automatically recorded on the VR device) and tracking of audio modules (minutes of use and number of modules used). VR and audio usage data were surveyed once every 4 weeks during the intervention period. Intervention use was also self-reported via the diary survey both groups were asked to complete at the end of each study day. Although participants were encouraged to use the modules (VR or audio) daily, there was no pre-determined, prescribed dose. While this approach facilitated an evaluation of individual variation in intervention usage, there was an inability to assess whether participants used the intervention to help mitigate pain or if usage was driven by their pain experience.

In the VR group, symptoms associated with cybersickness were measured (before and after VR use) with the Virtual Reality Sickness Questionnaires (VRSQs; see Table 2) then monitored throughout the intervention using the daily diary surveys and weekly check-ins by study staff. Based on prior findings [49,57], administration of the VRSQs was limited to during the first week of VR use to decrease participant burden and account for the likelihood of symptoms occurring most often during initial use. Cybersickness incidence was measured by the percentage of days with reported symptoms on the post-VR VRSQ.

Given the nature of SCD and individual disease experiences, it was anticipated that some participants might not complete all intervention modules or provide data at all study timepoints. Weekly check-ins with participants in both intervention groups were conducted by study staff via text messages, emails, and telephone calls to assess and encourage participants’ progress with the study activities, to inquire about any issues with the interventions or surveys that needed to be addressed by study staff, and any VR-associated symptoms not previously reported (e.g., if diaries were not completed).

### 2.5. Statistical Methods

Survey data were collected using the Research Electronic Data Capture (REDCap; Nashville, TN, USA) web-based application (versions 12–14) [79,80] and analyzed using SPSS (version 27) [60]. Descriptive statistics (mean, standard deviation, and frequency distribution) were used to assess demographic characteristics. All data collection timepoints (baseline, daily, week 4, week 8, and week 12) were used for analysis, and all measures were scored by their published protocol. Daily diary data were grouped into two time periods (1 [days 1–28] and 2 [days 29–56]) to better align with chronic pain-related data collected at weeks 4 and 8. Multi-level linear models (MLMs; a type of linear mixed-effects model) and generalized MLMs for binary outcomes, with group and time period as factors, were used to test for group, time, and group-by-time interactions. For each model, post hoc tests were performed to compare timepoints within groups and group differences at each timepoint using Sidak pairwise error rate corrections. Lastly, generalized estimating equations were used to compare symptom change scores across diary days completed. There were no family-wise error-rate adjustments for multiple simultaneous outcome measures because the study was not designed nor statistically powered for testing of composite outcome measures.

### 2.6. Qualitative Methods

A qualitative descriptive approach was also employed to provide contextual information to aid in interpreting the quantitative findings. A study goal was to conduct individual, semi-structured interviews about the intervention, after the intervention period, with approximately half of each study group (9 VR and 12 audio; 21 total), given that a sample size of 30 or less is adequate for reaching data saturation [81]. Of the study sample (*N* = 44), 24 individuals (8 VR and 16 audio) responded to the invitation (sent via their preferred contact method specified at baseline: text message, telephone call, and/or email) and agreed to participate in the interviews. The remaining individuals from the VR group declined the invitation to the interview because they discontinued the intervention (*n* = 3), declined the invitation with no reason provided (*n* = 1), or did not respond to the invitation (*n* = 7).

The principal investigator (PI) and two research staff (trained by the PI) separately contacted participants via the Zoom Workplace software program (Zoom, audio only) to conduct one interview with each participant from the trial selected via convenience sampling. The interviews, with only the interviewer and the interviewee present, were conducted using a semi-structured guide that was developed by the PI and pilot tested in a previous study of VR and chronic pain in SCD [56]. Interviewees were asked open-ended and closed-ended questions about (1) their experience of using the intervention, (2) their thoughts about the intervention, (3) any barriers to intervention use, and (4) any challenges while using the intervention. Interviewees’ responses to initial questions guided subsequent questions and probes were used to obtain details. The interviews (60 min average duration) were audio-recorded using Zoom, which automatically assigned sequential numbers to the interviews that were used for de-identification. A professional transcriptionist transcribed the audio-recorded interviews verbatim. Interviewees received an additional $50 gift card after participating in the interview.

#### 2.6.1. Interview Data Analysis

After the first two interviews were transcribed, the PI assessed the transcripts for accuracy before conducting an initial analysis of the transcripts and field notes concurrently with data collection. The probes for subsequent interviews were revised using emerging study findings. After 22 additional interviews were completed and transcribed, transcripts were assessed for accuracy and reviewed before the study team conducted content analysis. Concepts identified during initial data analysis were repeated during the remaining interviews. Because no new concepts were identified once all 24 interviews were completed, it was concluded that the findings adequately addressed the study objectives [82,83]. Therefore, it was determined that saturation was reached and no additional interviews were conducted.

Using a content analysis framework, codes were derived from the interview data, not a pre-determined codebook, and emerging patterns (in units of words, phrases, and sentences) were identified [84]. Analysis of the data included: (1) reading the transcripts to become familiar with the data as a whole and making notes of initial analysis; (2) identifying and quantifying words or content reflecting interviewees’ thoughts, concepts, or patterns as codes; (3) organizing codes into categories and identifying associated exemplars; and (4) summarizing the content. The PI and two research assistants individually conducted inductive coding of the transcripts. Subsequently, the study team reviewed findings of the individual analyses (codes, patterns of codes in the data, and data associated with each code) organized with Microsoft Word and Excel (Microsoft Corporation; Redmond, WA, USA; versions 2201–2502). Coded data were grouped and regrouped into study-related categories—participants’ experiences of using the intervention, their thoughts about the intervention, any barriers to intervention use, and any challenges while using the intervention—and differences in perspective among study team members were addressed until consensus was achieved [85]. The study team then refined and finalized the findings to draft the report [86]. The qualitative approach served to aid in interpreting the quantitative findings in this evaluation of the preliminary benefits of using EaseVRx versus audio attention control as in-home intervention for chronic pain self-management among adults with SCD.

#### 2.6.2. Rigor and Positionality

The following measures were employed to ensure rigor with respect to the qualitative methods. Interviews were conducted separately by the PI and two research staff. These research staff and the research assistants (who were involved in the qualitative data analysis) received qualitative training (including qualitative interviewing and coding) from the PI. During the interviews, informal member checking was utilized by asking interviewees to clarify their statements, explain their meaning, and validate study team members’ understanding of the data to ensure credibility [87,88,89,90]. This practice also aided in maintaining reflexivity and accurately representing participants’ voices. Interview transcripts were evaluated for similarities and differences across interviewees’ responses, and field notes were reviewed to glean any insight regarding participants’ experiences. All team members utilized the interview data analytic approach described, and there were discussions to understand perspectives of individual team members and achieve agreement regarding the findings.

The research staff who assisted in conducting the interviews are two female research interviewers (one Bachelor’s prepared and one Master’s prepared, with a combined four years of research interviewing experience). The PI is a female, PhD-prepared, academic nurse scientist with more than 12 years of expertise in SCD pain research, self-management, quantitative and qualitative methods, and interviewing adults with SCD, as well as five years of experience in remote delivery of VR interventions. Both research interviewers have personal knowledge and a family history of SCD, with one interviewer having sickle cell trait. Because these research staff were involved in interviewing participants enrolled from their own study site and some interviewees engaged in previous SCD research at the study site, interviewees may have had prior familiarity with their interviewer (the research staff or the PI). Other members of the study team are two female academic scientists: a nurse scientist (with more than 30 years of expertise in SCD community-engaged work, pain self-management and feasibility trials in SCD, and qualitative research) and a biostatistician (with more than 25 years of experience in conducting bio-behavioral healthcare research, evaluating community-based indices, and providing statistical support for clinical trials). Three study team members were involved in the qualitative data analysis: the PI and two female, Master’s degree program students working as research assistants (who had no prior qualitative research experience). Except for the statistician, who is White, all other study team members are individuals of African descent. However, the statistician had prior exposure to SCD through research collaborations with the PI. The identity and positionality of all study team members may have influenced the framing of interview questions, interviewees’ responses, and study findings and conclusions.

## 3. Results

**Figure 2:**
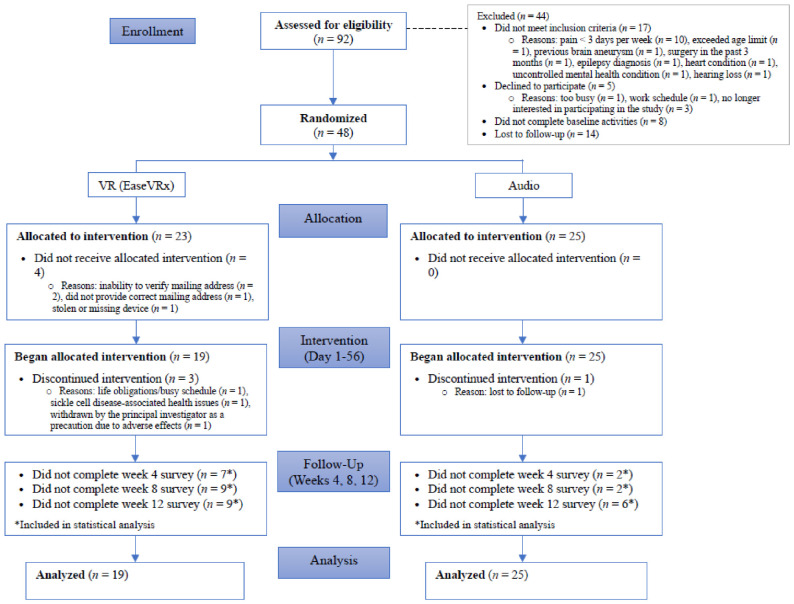
Study CONSORT flow diagram.

The CONSORT flow diagram for the trial, through 4 weeks after the treatment phase, is displayed in Figure 2. Of the 92 individuals who were assessed for eligibility, 75 were eligible for participation. Of the 75 eligible individuals, 93.3% (*n* = 70) were enrolled (lower than the pre-determined enrollment target of 72) and 58.7% (*n* = 44) participated in the study. After enrollment, 22 of the 70 enrollees (31.4%) either did not complete baseline study activities or were lost to follow-up. Although a total of 48 enrollees were evaluated for cybersickness risk (66.7%, *n* = 32 at low risk), randomized, and allocated to the interventions (23 VR and 25 audio), four were lost to follow-up. Of the 44 participants, 23 (52.3%) were recruited remotely. Of those recruited remotely, five were randomized to VR and 18 were randomized to audio based on the randomized block design that was stratified by biological sex.

Because the final number of individuals consented and randomized fell short of the goal, revised power calculations were conducted, using SPSS (version 27) [60], which indicated that a sample size of 44 would enable detection of a large effect size difference (Cohen’s *d*) of 0.87 with adequate power (80%) at a 5% significance level. In accordance with intent to treat principles, analyses included all participants who successfully completed baseline activities and began the allocated intervention (19 VR and 25 audio) (see Figure 2), although they may not have provided data at all the post-baseline assessment timepoints (week 4, week 8, and week 12).

### 3.1. Participants’ Demographic, Clinical, and Pain Characteristics

Participants’ baseline demographic, clinical, and chronic pain characteristics are provided in Table 3. The 44 study participants were 22–48 years of age (average 33.73 years; standard deviation [SD] 7.50). Most of the participants identified as Black or African American (93.2%, *n* = 41), non-Hispanic/non-Latino (95.5%, *n* = 42), and female (61.4%, *n* = 27). Most were single/never married or in a domestic partnership (68.2%, *n* = 30), had at least some college education (86.4%, *n* = 38), and were currently employed (52.3%, *n* = 23). Of the participants, 52.3% (*n* = 23) reported an annual income of between $25,000 and $99,999, and 56.8% (*n* = 25) reported their income source as wages/salary. Most participants had a SCD type of hemoglobin SS (Hb SS), known as sickle cell anemia (70.5%, *n* = 31), a medium or high severity of SCD (70.5%, *n* = 31), and a chronic pain grade classification of Grade III (high disability–moderately limiting) or Grade IV (high disability–severely limiting) (65.9%, *n* = 29). Additionally, 81.8% (*n* = 36) of the participants reported experiencing fatigue/low energy (on an average of 4.5 days [SD 1.7, range of 2–7] during a typical week) and almost half (43.2%, *n* = 19) reported trouble dealing with stress (most attributed to finances/bills and pain).

Across participants, 1769 diary days were completed (71.8% of a possible 2464 total diary days). On 45.4% of those days (*n* = 804), participants described the type of pain that they experienced. Non-crisis chronic pain, regular pain, or everyday pain was reported on 569 diary days (71%). Acute pain was reported on 187 diary days (23.3%). Pain crisis was reported on 225 diary days (28%), and “recovering from crisis/last crisis pain” was reported on two diary days (0.25%). Most of the participants experienced chronic, non-crisis pain at a frequency of either 7 days (35%, *n* = 14) or 3 days (27.5%, *n* = 11) during a typical week. On a scale from 1 to 10, the average daily pain score on those days was 5.78 (range from 2 to 10). Chronic pain was felt in multiple body locations (86.4%, *n* = 38) and the most common locations were the back (88.6%, *n* = 39; primarily lower back), legs (47.7%, *n* = 21), and hip (34.1%, *n* = 15)). On most of the diary days completed, participants described their pain as aching (27.6%, *n* = 488 diary days) or throbbing (18.9%, *n* = 334). The most common source of the pain in the preceding 12 h was reported as cold temperature (15.9%, *n* = 282), overexertion or doing too much activity (14.9%, *n* = 264), or stress (12.5%, *n* = 221).

To help with pain, participants used medication strategies and non-medication strategies. Medications used most often for pain were Morphine sulfate extended release (*n* = 189 diary days), Morphine (*n* = 165 diary days), and Oxycodone (*n* = 156 diary days), with most participants using one tablet (*n* = 123 diary days) or two tablets (*n* = 114 diary days). No medication was used on 151 diary days. Non-medication strategies used most often for pain were distraction (*n* = 479 diary days), warmth/heat (*n* = 470 diary days), intake (e.g., drinking water, healthy diet, or natural foods/drinks; *n* = 291 diary days), cognitive reframing/relaxation (*n* = 223 diary days), and dealing with emotional or physical stress (*n* = 176 diary days). Most participants used these strategies twice (*n* = 149 diary days) or three times (*n* = 134 diary days) during a given day.

### 3.2. Study Outcomes

Across both intervention groups, questionnaire response was 100% (*N* = 44) at baseline, 79.5% (*n* = 35) at week 4, 75% (*n* = 33) at week 8, and 65.9% (*n* = 29) at week 12. Survey non-completion rates and reasons at post-baseline assessment timepoints are provided in Table 4. The rate of completion of all four post-baseline assessment timepoints was 47.4% (*n* = 9) in VR and 72% (*n* = 18) in audio; however, there was no statistically significant difference (*p* = 0.096). Most of the missing data was due to attrition over time, while some of the missing data was due to intermittent missingness. Two participants remained in the study through week 12 although they did not provide data at all four timepoints. Consequently, 29 participants had data at week 12 though only 27 had data at all four timepoints. There was no statistically significant difference between the two randomization groups nor between participants who did and did not provide data at all four timepoints with respect to age, sex, education, marital status, or income. However, participants who were working (paid or unpaid) were more likely to have data at all four timepoints (*p* = 0.002).

Participants used the intervention modules for an average of 19.1 and 12.1 min per day in VR and audio, respectively. The sample size and lack of data granularity limited the ability to identify specific usage patterns across the two daily pain diary time periods (1 [days 1–28] and 2 [days 29–56]). However, the number of days on which participants used the intervention one or more times increased slightly over time in VR (from 54.4% during time period 1 to 59.5% during time period 2; *p* = 0.649) while significantly decreasing over time among participants in the audio group (from 71.6% during time period 1 to 62.8% during time period 2; *p* = 0.006). Feedback from interviewees about the interventions (barriers to use, challenges while using, positive aspects, and negative aspects), which may offer reasons for usage patterns, is also provided in Table 4.

**Figure 3:**
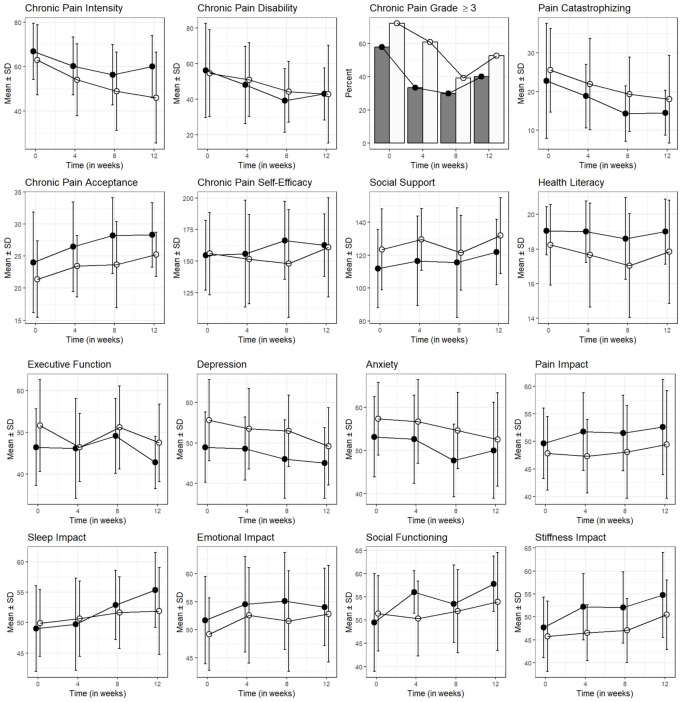
Outcomes by timepoint and group. This figure displays the plots of the means ±1 standard deviation [SD]) of the scores on the outcome measures by timepoint (baseline through week 12) and group, and the percentage of participants with a high (≥Grade 3) chronic pain grade classification for each timepoint and group. Means are displayed as circles with error bars showing +1 and −1 SD above and below each mean. The black filled circles (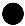) signify the virtual reality group and the white circles (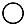) signify the audio group.

Pain levels in both groups decreased from time period 1 to 2 (*p* < 0.001), with a reduction of 1.42 points in VR and 0.54 points in audio (*p* = 0.056). As shown in Figure 3 (also see Table S1), the plots indicate trends in outcome changes that suggest signal of benefit and partially support the study hypothesis: preliminary benefits of EaseVRx will include decreased pain; improved pain coping, sleep, and functional ADLs; decreased disability; and enhanced QoL. Between baseline and week 12, significant improvements were seen for pain intensity (*p* = 0.002), pain catastrophizing (*p* = 0.005), and chronic pain acceptance (*p* = 0.002) in both groups. For each of these outcomes, the group-by-time effects were not statistically significant due to the smaller sample sizes (*p* = 0.980 for chronic pain acceptance, *p* = 0.455 for pain intensity, and *p* = 0.983 for pain catastrophizing). The effect size estimates for the changes from baseline to week 12 for each of these outcomes by group are: (1) chronic pain acceptance: Cohen’s *d* = 0.337 for VR and *d* = 0.433 for audio; (2) pain intensity: Cohen’s *d* = −0.105 for VR and *d* = −0.747 for audio; and (3) pain catastrophizing: Cohen’s *d* = −0.506 for VR and *d* = −0.629 for audio. Higher VR usage (number of modules used) was associated with larger decreases in pain intensity levels from baseline to week 8 (Spearman’s rho = −0.786, *p* = 0.036).

There were no statistically significant differences between groups for chronic pain disability, chronic pain self-efficacy, anxiety, social support, pain impact, emotional distress, or social functioning. However, in the VR group, sleep impact scores improved (*p* = 0.009) and social functioning scores increased slightly (*p* = 0.080). In the audio group, there were improvements in depression (*p* = 0.045), stiffness impact (*p* = 0.026), and executive function (*p* = 0.001). Higher VR usage was negatively associated with the number of medications used for pain (slope = −0.018, *p* = 0.019). For audio, the number of medications used for pain slightly increased over time (*p* = 0.023).

There were 48 diary days across 17 VR participants with completed VRSQs, of 119 total possible diary days on which VRSQs were administered. Participants reported worsened post-VR general discomfort on 6 days (12.5%), headache on 5 days (10.4%), and fatigue on 4 days (8.3%). However, there were significant improvements in post-VR general discomfort on 18 days (37.5%, *p* = 0.012), fatigue on 12 days (25%, *p* = 0.033) and difficulty focusing on 7 days (14.6%, *p* = 0.070). There was a significant reduction in scores for symptoms associated with cybersickness, with an average change from pre-VR to post-VR timepoints of 0.88 points (95% confidence interval [CI] from −1.52 to −0.23). One VR participant experienced nausea and dizziness that did not require medical intervention, and they wanted to continue using the VR device. However, in accordance with the safety protocol established before trial initiation, the participant was withdrawn from the study by the PI to prevent any potential worsening of symptoms.

### 3.3. Interviewee Feedback About the Interventions

Content analysis of the open-ended and close-ended questions asked during the 24 interviews found that, overall, the interventions were acceptable to the interviewees. Example interview questions and interviewees’ quotes are provided in Table S2. On a 4-point Likert scale (strongly agree, somewhat agree, somewhat disagree, strongly disagree), the median scores in both groups were at the highest level of 4 for these questions: (1) The VR/audio program was easy to use and (2) I enjoyed using the VR/audio program. Interviewees described the interventions as “helpful,” “relaxing,” “interesting,” and “insightful”. For both groups, the most positive aspects of the interventions (see Table 4) were that they helped with managing pain, had appealing program content, helped with relaxation, and were calming. However, interviewees in both groups reported barriers to intervention use and challenges while using the intervention that are presented in Table 4. The most common barriers for both groups were time constraints/competing life demands (e.g., work), “high” sickle cell pain, distractions in the home environment, and fatigue. For both groups, technical glitches were the most common challenge experienced while using the interventions. Additionally, for both groups, the most negative aspect of the interventions was content repetition. Nonetheless, of the 24 interviewees, 87.5% (*n* = 21) reported being satisfied with the intervention, 79.2% (*n* = 19) that it was acceptable for coping with their chronic pain, and 100% reported a high likelihood (7–10 on a scale from 0 to 10) of recommending it to someone else with SCD who has chronic pain.

## 4. Discussion

This exploratory, pilot randomized trial demonstrated mixed preliminary benefits of an in-home VR intervention on chronic pain and chronic pain correlates in adults with SCD. Both interventions were perceived by participants as beneficial for pain management and relaxation. VR was associated with decreased chronic pain intensity, which was clinically significant [64] and greater than in audio, with higher VR usage being associated with larger decreases in pain intensity and fewer medications used for pain than in audio. VR was also associated with improved pain coping (pain catastrophizing and chronic pain acceptance), sleep impact, and social functioning. Notably, benefits for chronic pain intensity and pain coping persisted through 4 weeks post-intervention. These findings are consistent with a prior study of EaseVRx among individuals with chronic lower back pain in which VR improved pain intensity and sleep interference, and over-the-counter analgesic use decreased [49]. Because high rates of pain catastrophizing [13,56] and challenges with sleep [20] and social functioning [91,92] occur among adults with SCD, potential VR benefits in these areas are promising.

Unexpectedly, VR benefits for chronic pain self-efficacy, anxiety, depression, functional ADLs, disability, and QoL were not supported. This differs from a prior study of EaseVRx in chronic lower back pain where VR improved physical function and pain-related interference with activity, mood, and stress [49]. Similar results may not have been achieved in the current study because (1) there was widespread pain among participants; (2) the sample size was small, and the study was only powered to detect large effect sizes; and (3) participants’ use of the intervention across the study duration may not have been sufficient for changing these outcomes. Future studies should evaluate whether VR may be more beneficial for some adults with SCD compared to others.

Although scores for symptoms associated with cybersickness significantly decreased from pre-VR to post-VR timepoints, symptoms were reported. Potential contributors to cybersickness include older age, female sex, less experience with VR content, use of virtual controls rather than physical navigation, use of a head-mounted display, higher level of immersive content, and longer duration of exposure [45]. In this study, the delivery of highly immersive content to mostly female participants via a headset with virtual controls may have contributed to the reported symptoms. Notably, because symptoms were present prior to and following VR use, symptoms could be independent of VR. Nonetheless, symptoms did not appear to hinder program use. Further research is needed to evaluate the mixed results regarding symptoms associated with cybersickness and determine how to best prevent or mitigate symptoms, such as incorporating 10–15 min breaks after 15–30 min of VR use and lowering the brightness of the headset [93]. More research is also needed to understand what factors contribute to adults with SCD experiencing symptoms associated with cybersickness in the absence of VR and in what ways those symptoms can be addressed.

Like VR, audio was associated with improvements in chronic pain intensity and pain coping that persisted through 4 weeks post-intervention. Audio was also associated with improvements in depression, stiffness impact, and executive function. These findings should be investigated further because challenges with cognitive dysfunction [13,56,91] and depression [14,20,91] occur among adults with SCD who experience chronic pain. Along with stiffness impact, these factors may be useful to consider when evaluating and addressing chronic pain. Increased use of medications for pain over time in the audio group could indicate less benefit of audio compared to VR and could have contributed to improvements in chronic pain intensity. Future research is needed to understand these findings.

Adults with SCD in the U.S. have been considered as a hard-to-reach population for conducting clinical trials. Along with challenges to research participation (such as logistics) faced by Black or African American individuals in the U.S. [94], engagement in self-care and prior healthcare or research experiences can influence research participation among adults with SCD [95]. In the current trial, 92 individuals were reached and 81.5% were eligible for participation, which is consistent with previous reports of the prevalence of chronic pain among adults with SCD [13]. Although 93.3% of the eligible individuals were enrolled, 31.4% of the enrollees either did not complete baseline activities or were lost to follow-up. These occurrences are common in studies of individuals with SCD, as they experience an enormous illness burden and severe disease complications [10,12]. It is noteworthy that in the current trial, although enrollment was slower and lower than anticipated, higher recruitment and retention rates were achieved across both intervention groups than in a prior study of adults with SCD [57]. These findings suggest improved engagement strategies, enhanced communication, and perhaps greater receptivity to non-pharmacological interventions among adults with SCD.

The median duration of intervention use across the trial was less than half of the program duration. This finding is consistent with clinical trials of digital interventions wherein 44.2% of participants typically complete all treatment modules [96]. Additionally, less engagement is often found among minority populations [97]. The barriers to intervention use, challenges during intervention use, and negative aspects of the interventions reported by the interviewees may have contributed to decreased adherence over time. Reported factors such as time constraints and competing life demands, sickle cell pain, and feeling unwell or being hospitalized can affect engagement of adults with SCD in these types of intervention studies, so these factors should be proactively addressed using insight from the target population. Technical glitches, which could be associated with technical failures or internet connectivity challenges, are not uncommon in technology-based behavioral interventions. In future work, researchers should ensure that participants can consistently access the internet, receive adequate training on utilizing study technology, and are provided with enhanced support for navigating technical issues. Content repetition, which was perceived as a negative aspect of the interventions, is an important technique for learning. Both interventions, with program content including pain education, were designed to aid participants in gaining knowledge about pain and pain coping, and to facilitate their acquisition and development of pain self-management skills. Therefore, content was reinforced by providing varied opportunities for practicing skills. Despite challenges to engagement, participants’ use of the intervention for at least 12 min per day in both groups is a promising finding. In future studies, it may be beneficial to incorporate additional strategies (such as modifying program content, tailoring financial compensation schedules, and employing patient navigation services) to increase VR use. Health coaching, a patient-centered process incorporated into care delivery wherein trained health coaches guide and support individuals in achieving personal health outcomes [98], may be helpful in facilitating exposure of adults with SCD to VR as a non-pharmacological pain management strategy and encouraging its use for the full duration of intervention programs. In a future larger trial, it may also be informative to explore the influence of participant characteristics on the relationships among VR use and study outcomes.

In addition to almost half of the participants reporting trouble dealing with stress at baseline, throughout the study, participants reported stress as one of the most common sources of their pain and noted dealing with emotional or physical stress as one of the non-medication strategies they used most often for pain. The VR and audio modules incorporate cognitive-behavioral training to reframe thoughts about pain and build coping strategies. Given the potential benefits of this and other intervention content for stress and pain, in future research, it may be useful to include an evaluation of biomarkers that are applicable to chronic pain in SCD (such as soluble E-selectin) [99] and are associated with stress (such as oxidative stress markers [e.g., reactive oxygen species] and stress–pain interaction biomarkers [e.g., cortisol and psychosocial stress]) [100,101].

Study findings are relevant for adults with SCD in the U.S. and in other countries, given the global pattern of SCD-related need [5,6] and shared challenges in accessing behavioral pain management strategies [37,38,39]. Considering the limited availability of behavioral interventions/services to address chronic pain and the need for available resources to support multimodal pain management, study findings contribute initial insights regarding the preliminary benefits of non-pharmacological interventions like VR and audio pain management and the potential for their integration into SCD care in inpatient, outpatient, and community-based settings. Actionable pathways for integration may include: (1) facilitating participation of adults with SCD in research studies needed to further evaluate the benefits of non-pharmacological strategies for health outcomes and their integration into SCD care; (2) offering the interventions to patients or patients requesting the interventions, during healthcare visits, as self-management strategies for pain coping and relaxation that can be used in healthcare settings and at home; (3) working together with healthcare providers to incorporate these strategies into individualized SCD pain plans as a complement to clinical care; and (4) partnering with community-based organizations to increase access to these strategies. One approach for fostering the development of integrated care models may be health coaching management. Health coaching management can be conducted in collaboration with healthcare providers and community-based organizations to inform the design, evaluation, and system integration [102] of these types of non-pharmacological interventions to help enhance self-care behaviors, address psychosocial factors, improve engagement and adherence to self-management tools, and influence long-term outcomes in this population.

### 4.1. Strengths

There were notable strengths of this study that can inform future research. Among adults with SCD who experience chronic pain, the trial aimed to evaluate the use of an in-home VR pain management program that was developed for chronic lower back pain. A randomized design, an attention control comparator, validated pain and psychosocial instruments, and multiple assessment timepoints were incorporated into the study. To better understand real-world implementation of VR, study participants had the autonomy of utilizing the intervention at times that best fit their pain management needs, daily lives, and self-management goals.

### 4.2. Limitations

The strengths of this study should be considered in the context of several limitations. This trial was conducted, using an in-home VR pain management program that was developed for chronic lower back pain, in a small sample of adults with SCD that consisted of a high proportion of females, individuals with medium or high SCD severity, high chronic pain grade classification, and those with a college degree who were currently employed. Future studies should seek a larger, more diverse sample and explore differences by participant characteristics. The dropout rate after enrollment, particularly among males and individuals randomized to VR, contributed to the difference in size of the intervention groups, introduced an attrition bias, and resulted in challenges to power. There was also sporadic completion of the daily diary among some participants and the rate of non-completion of surveys in the VR group exceeded the estimated 20% that was anticipated. Given the pilot nature of this trial, there were limitations regarding including additional variables in each model for covariates adjustment. However, the differences in attrition and missing data between the two randomization groups were not statistically significantly different. The use of usual pain management strategies during the trial may have contributed to varying levels of engagement and improvements in study outcomes in both groups. Differences among participants who did or did not complete all study timepoints were investigated, but the models were not adjusted further for working status. Because this trial was conducted outside of the healthcare setting and relied on participants’ unconfirmed self-report from a portion of the sample, some data may also be subject to social desirability bias and challenges with recall. As some adults with SCD in the U.S. interact with healthcare systems infrequently if at all, there was value in recruiting and engaging with individuals outside of those settings although that approach inherently limited the ability to objectively confirm SCD status. Chronic pain and chronic pain-related surveys were not administered immediately before and after daily use of the interventions. Additionally, while pain education is a program component, a pre- and post-intervention survey about pain knowledge was not administered to participants. Although VR users were instructed to complete the VRSQ before and after using the VR intervention at home, the rate of completion of VRSQs was 40.3% and some participants may have completed initial pre-VR VRSQs after using the intervention, which may have contributed to higher scores for symptoms associated with cybersickness on the pre-VR VRSQs than on the post-VR VRSQs. Also, because VR users may have acclimated to the VR environment, their scores on the post-VR VRSQs could suggest that they experienced fewer symptoms associated with cybersickness than they expected to experience. VR might have different benefits for individuals with a different chronic pain and SCD experience, and history of exposure to non-pharmacological pain management therapies, than participants in this study. Given these limitations, the study results are not generalizable to the overall population of adults with SCD.

It is important to note that although the current trial focused on chronic pain, the potential of acute pain and pain crises being influential factors cannot be negated. There may have been days on which acute pain and pain crises were experienced but not reported, and participants’ experience of acute pain and pain crises may have influenced completion of outcome measures and intervention use. Because study participants had autonomy in engaging with the interventions, intervention use was driven by their choice, pain experiences (e.g., chronic pain levels and acute pain episodes), and need for addressing pain. For instance, individuals with either higher levels of pain or more acute pain episodes were more likely to use the interventions to address pain. Daily use of medication and non-medication strategies was self-reported and may also have correlated with the incidence of acute pain episodes. Acute pain episodes may have contributed to high or low use of the interventions and medications, and they may have influenced the outcome scores. Further research is needed to understand these findings in the context of the potential benefits of VR for both acute and chronic pain in SCD in the home setting. This is important because of evidence supporting that VR is feasible, acceptable, and safe in hospitalized patients with SCD among whom immersive VR significantly reduced pain intensity and the number of painful body areas while experiencing pain episodes [103]. In addition, researchers should consider powering future studies of in-home VR in SCD for multiple outcome measure testing and include prescriptive protocols for more routine VR usage, with use also on days when pain is not experienced, to understand how routine use of VR affects pain management prospectively.

## 5. Conclusions

Although preliminary, the current research adds to the limited studies that explore VR for chronic pain. The findings highlighted the feasibility of using in-home VR for chronic pain and suggest benefits for chronic pain and chronic pain correlates. Therefore, VR has the potential to be employed as an in-home tool to positively mitigate some of the pain management challenges experienced by adults with SCD. The study results can serve as a baseline for future investigations that are necessary for detecting changes in outcomes among adults with SCD who are offered VR as a chronic pain self-management intervention. Future fully powered trials are needed to understand the preliminary findings of this study, evaluate VR treatment effects over time, and assess the effectiveness of VR when compared to other chronic pain management strategies, particularly in the home setting. This additional research can help to inform the implementation of VR-based interventions and their integration into clinical care to address chronic pain management disparities in the SCD community. To strengthen those future trials, researchers should seek to obtain larger sample sizes, reduce attrition, incorporate strategies to address symptoms associated with cybersickness, assess how to best incorporate intervention use into the daily lives of adults with SCD, and include measurement of biomarkers (e.g., of stress, inflammation, or pain modulation) to further evaluate the benefits of the interventions.

## Figures and Tables

**Table 1:** Participant inclusion and exclusion criteria.

**Inclusion Criteria** Age 18–50 years. Diagnosis of sickle cell disease. Chronic non-vaso-occlusive pain experienced ≥3 days per week on average for >6 months. Chronic non-vaso-occlusive pain was differentiated from vaso-occlusive pain (VOC, or pain crisis) based on the pattern (ongoing daily or near-daily pain), time course (duration > 6 months), patient history (pain occurring between crises and pain being different from crisis pain), associated findings (pain descriptors used and pain being less responsive to typical VOC treatment), and functional impact [17,18,34]. Ability to wear a virtual reality (VR) head-mounted display and move head in cervical rotation, extension, and flexion. Ability to operate VR device equipment such as buttons on a headset or an external remote controller. Ability to read, write, and understand English.
**Exclusion Criteria** Conditions: Co-morbidities (such as neurological, psychosocial, or other disorder) that may influence pain perception. Other disorders include autoimmune and inflammatory disorders (e.g., lupus), endocrine and metabolic disorders (e.g., diabetic neuropathy), or central nervous system disorders (e.g., fibromyalgia). Diagnosis of epilepsy or susceptibility to seizures, migraines, or other neurological disorders that may prevent VR use, or other medical conditions predisposing individuals to nausea and dizziness. Susceptibility to claustrophobia, motion sickness or cybersickness (digital motion sickness). History of blackouts. Hypersensitivity to flashing lights or motion. Lack of stereoscopic vision. Severe visual or hearing impairment. Inability to operate VR equipment (such as inability to turn head or use hands to operate external remote controller). Injury to the eyes, face, head, or neck that prevents comfortable VR device use. Other exclusions: Medical instability as determined by healthcare provider. Significant motor impairment. Surgery within the past three months. Plan to begin a new pain management strategy (such as medication, physiotherapy, acupuncture, or cognitive behavioral therapy) in the succeeding three months. History of major psychiatric disorder (such as schizophrenia or bipolar disorder) not controlled with medication or behavioral factors that would interfere with study procedures. Alcohol or substance dependence, heart conditions, or the presence of implanted medical devices (such as cardiac pacemakers). Cognitive or developmental disabilities. Active suicidal ideation. Inability to read, write, or understand English. Pregnancy (because it was not yet known whether VR has effects on the fetus). Plans for vacation in the succeeding three months.

**Table 2:** Description, administration, and reliability of study measures.

Measure	Data Collected and Validity (Where Applicable)	Administration and Reliability in the Study (at Baseline)
Demographic Questionnaire	Characteristics including age, sex and gender identity, sickle cell disease type, annual income, years of education, number of chronic pain days, and daily pain rating. This questionnaire was used in previous studies of adults with sickle cell disease [13,14,56,57].	Baseline Cronbach’s alpha (Cα) is not applicable
Adult Sickle Cell Quality of Life Measurement Information System (ASCQ-Me) Sickle Cell Disease Medical History Checklist	Sickle cell disease severity based on sickle cell-associated health complications, including hip or shoulder damage [64]. Low (scores < two), medium (score of 2), or high (scores > two) severity. In previous studies, this checklist was established as a valid indicator of sickle cell disease severity among adults [64,65]. The checklist was used in previous studies of adults with sickle cell disease [13,56,57].	Baseline Cα is not applicable
Risk for Cybersickness Screening Form	Cybersickness risk factors (within ≥10 years) including migraine propensity, motion sickness history, prior negative experiences during virtual motion exposure (e.g., scrolling rapidly on a smartphone), and exposure to conditions associated with poor balance (e.g., vertigo) [66]. Low risk is ≤2 risk factors. This screening form was used in previous studies of adults with sickle cell disease [56,57].	Baseline Cα is not applicable
Chronic Pain Grade Questionnaire	Chronic pain grade based on chronic pain intensity and chronic pain disability: Grade 0, no intensity–no disability; Grade I, low intensity–low disability; Grade II, high intensity–low disability; Grade III, high disability–moderately limiting; and Grade IV, high disability–severely limiting [67]. In previous studies, evidence of validity included significant correlations between the questionnaire and the Short Form (SF)-36 [67]. This questionnaire was used in previous studies of adults with sickle cell disease [13,56,57].	Baseline, Week (W) 4, W8, and W12 Chronic pain intensity Cα = 0.575 Chronic pain disability Cα = 0.920
Pain Catastrophizing Scale	Pain catastrophizing: Thoughts and feelings when participants experienced pain [68]. The scale was used in previous studies of adults with sickle cell disease [13,56,57].	Baseline, W4, W8, and W12 Cα = 0.951
Chronic Pain Acceptance Questionnaire-8	Acceptance of chronic pain [69]. The questionnaire was used in previous studies of adults with sickle cell disease [13,56,57].	Baseline, W4, W8, and W12 Cα = 0.700
Chronic Pain Self-Efficacy Scale	Efficacy expectations for coping with the consequences of chronic pain [70]. This scale was used in previous studies of adults with sickle cell disease [13,56,57].	Baseline, W4, W8, and W12 Cα = 0.925
BRIEF Health Literacy Screening Tool	Help needed by participants in healthcare situations [71]. Health literacy levels: Inadequate (scores of 4–12), marginal (scores of 13–16), and adequate (scores of 17–20) [71]. This tool shows evidence of convergent validity with the Short-Test of Functional Health Literacy in Adults questionnaire (*r* = 0.42) and the Rapid Estimate of Adult Literacy in Medicine test (*r* = 0.40) [71]. The tool was used in previous studies of adults with sickle cell disease [13,56,57].	Baseline, W4, W8, and W12 Cα = 0.702
Behavior Rating Inventory of Executive Function—Adult Version (BRIEF-A)	Participants’ views of their executive functions or self-regulation in their everyday environment [72]. Reliability and validity are well-established for the BRIEF. While the BRIEF has been used in children and adolescents with sickle cell disease [73], limited evidence exists regarding the use of BRIEF-A in sickle cell disease.	Baseline, W4, W8, and W12 Cα = 0.963
Patient-Reported Outcomes Measurement Information System (PROMIS) Anxiety 8a—Adult Version 1.0	Self-reported fear (fearfulness and panic), anxious misery (worry and dread), hyper-arousal (tension, nervousness, and restlessness), and somatic symptoms related to arousal (racing heart and dizziness) [74]. Scores are categorized as mild (T scores of 55–60), moderate (T scores of 60–70), and severe (T scores > 70). The validity of PROMIS measures has been supported, and compared to ASCQ-Me, for use in adults with sickle cell disease [64].	Baseline, W4, W8, and W12 Cα = 0.935
PROMIS^®^ Depression 8a—Adult Version 1.0	Self-reported negative mood (sadness and guilt), views of self (self-criticism and worthlessness), and social cognition (loneliness and interpersonal alienation), as well as decreased positive affect and engagement (loss of interest, meaning, and purpose) [74]. Scores are categorized as mild (T scores of 55–60), moderate (T scores of 60–70), and severe (T scores > 70). The validity of PROMIS measures has been supported, and compared to ASCQ-Me, for use in adults with sickle cell disease [64].	Baseline, W4, W8, and W12 Cα = 0.952
Social Support Questionnaire	Perceptions of the desirability, availability, use, and usefulness of social support [75]. In previous studies, evidence of validity included significant correlations between this questionnaire and the Commitment subscale of Kobasa’s Hardiness Scale [75]. This questionnaire was used in previous studies of adults with sickle cell disease [13,56].	Baseline, W4, W8, and W12 Cα = 0.957
ASCQ-Me Short Forms: Sleep Impact, Pain Impact, Emotional Impact, and Stiffness Impact	Sleep disturbances, the effects of sickle cell disease pain on activities of daily living, the effects of sickle cell disease on emotional well-being, and joint stiffness during the previous seven days [76]. The validity of ASCQ-Me Short Forms has been supported, and compared to PROMIS measures, for use in adults with sickle cell disease [64,76].	Baseline, W4, W8, and W12 Cα = 0.840 Cα = 0.900 Cα = 0.838 Cα = 0.864
ASCQ-Me Short Form: Social Functioning	The influence of health on social functioning during the previous 30 days [76]. The validity of ASCQ-Me Short Forms has been supported, and compared to PROMIS measures, for use in adults with sickle cell disease [64,76].	Baseline, W4, W8, and W12 Cα = 0.913
ASCQ-Me Short Form: Pain Episode Frequency and Severity Measure	The frequency and severity of sickle cell disease pain episodes during the previous seven days to the previous 12 months [76]. The validity of ASCQ-Me Short Forms has been supported, and compared to PROMIS measures, for use in adults with sickle cell disease [64,76].	Baseline, W4, W8, and W12 Cα is not applicable
Virtual Reality (VR) Sickness Questionnaire	Symptoms associated with VR sickness/cybersickness [77]. Two components: Oculomotor (general discomfort, fatigue, eye strain, and difficulty in focusing) and disorientation (headache, fullness of head, blurred vision, dizziness with eyes closed, and vertigo) [77]. Scale from 0 (none) to 3 (severe). This questionnaire was used in a previous study of adults with sickle cell disease [57].	Before and after VR use daily; post-baseline to the end of W1 Cα is not applicable
Pain Diary Survey (revised version of survey previously used by individuals with sickle cell disease) [78]	Survey questions focused on the following topics: (1) Daily pain and self-management: pain intensity (using the Numerical Pain Rating Scale), pain location, pain description, source of pain, pain interference (effect of pain on sleep the previous night, activities during the day, interactions with friends and family during the day, and mood during the day), and treatment (medication strategies used and amount used, as well as non-medication strategies used and frequency of use, during the day). For example, the question about pain intensity was “Click the number that shows how much overall PAIN or HURT you experienced today. (Range from 0 = No pain to 10 = Worst pain)” and the question about pain location was “Where on your body did you experience PAIN or HURT today? (List all the areas)”. (2) Intervention use: Total minutes of intervention use during each day. (3) Cybersickness: Any VR-associated nausea. This diary was developed and validated among adolescents and young adults with sickle cell disease [78] and was used in a previous study of adults with sickle cell disease [57].	Daily, post-baseline to W8 Cα is not applicable

**Table 3:** Participants’ baseline demographic, clinical, and chronic pain characteristics.

Measure	Category	All (44) *N* (%)	Virtual Reality (19) *N* (%)	Audio (25) *N* (%)
Age	22–30	20 (45.5)	5 (26.3)	15 (60)
	31–40	13 (29.5)	9 (47.4)	4 (16)
	41–48	11 (25)	5 (26.3)	6 (24)
Sex and gender identity	Born female/Identify as female	27 (61.4)	12 (63.2)	15 (60)
	Born male/Identify as male	16 (36.4)	7 (36.8)	9 (36)
	Born female/Identify as male	1 (2.3)	0 (0)	1 (4)
Race	Black or African American	41 (93.2)	17 (89.5)	24 (96)
	American Indian or Alaska Native	2 (4.5)	1 (5.3)	0 (0)
	Other (“Latino/Caribbean Native Indian”)	1 (2.3)	0 (0)	1 (4)
Ethnicity	Non-Hispanic/Non-Latino	42 (95.5)	19 (100)	23 (92)
	Hispanic/Latino (Puerto Rican and Central or South American)	2 (4.5)	0 (0)	2 (8)
Country of origin	United States of America/USA	40 (90.9)	17 (89.5)	23 (92)
	Ghana	1 (2.3)	0 (0)	1 (4)
	Nigeria	1 (2.3)	1 (5.3)	0 (0)
	Jamaica	1 (2.3)	1 (5.3)	0 (0)
	Haiti	1 (2.3)	0 (0)	1 (4)
Education	High school junior (11th grade)	1 (2.3)	1 (5.3)	0 (0)
	High school graduate or equivalent	5 (11.3)	4 (21.1)	1 (4)
	Some college, no degree	12 (27.3)	5 (26.3)	7 (28)
	Associate degree	3 (6.8)	1 (5.3)	2 (8)
	Bachelor’s degree	22 (50)	7 (36.8)	15 (60)
	Master’s degree	1 (2.3)	1 (5.3)	0 (0)
Employment status	Working, paid or unpaid	23 (52.3)	6 (31.6)	15 (60)
	Disabled, permanently or temporarily	15 (34.1)	8 (42.1)	7 (28)
	Unemployed, looking for work	4 (9.1)	3 (15.3)	1 (4)
	Homemaker	1 (2.3)	1 (5.3)	0 (0)
	Other (Patient advocate)	1 (2.3)	0 (0)	1 (4)
Marital status	Never married (single)	15 (34.1)	10 (52.6)	5 (20)
	Domestic partnership/living with partner/committed relationship	15 (34.1)	5 (36.3)	10 (40)
	Married	11 (25)	3 (15.8)	8 (32)
	Separated or divorced	2 (4.6)	0 (0)	2 (8)
	Widowed	1 (2.3)	1 (5.3)	0 (0)

**Table 4:** Post-baseline survey completion and intervention feedback.

**Post-Baseline Survey Completion**		
**Outcome**	**Virtual Reality (*N* = 19)**	**Audio (*N* = 25)**
Daily diary survey	18 participants (94.7%) completed daily diaries, ranging from 1 to 55 diaries (mean of 28), for a total of 691 diary days (64.9% response)	24 participants (96%) completed daily diaries, ranging from 1 to 55 diaries (mean of 32), for a total of 1078 diary days (77% response)
Week 4 questionnaires	*n* = 12 (63.1% response)	*n* = 23 (92% response)
Week 8 questionnaires	*n* = 10 (52.6% response)	*n* = 23 (92% response)
Week 12 questionnaires	*n* = 10 (52.6% response)	*n* = 19 (76% response)
Reasons for survey non-completion across timepoints	Busy schedule (*n* = 2), sickle cell pain treatment (*n* = 1), forgetting to complete surveys (*n* = 1), discontinued the intervention (*n* = 3), and no reason provided (*n* = 5)	Busy schedule (*n* = 2), emails about surveys were sent to junk email folder (*n* = 1), discontinued the intervention (*n* = 1), and no reason provided (*n* = 4)
**Intervention Feedback**		
**Topics of Interview Questions**	**Virtual Reality (*N* = 8)**	**Audio (*N* = 16)**
Barriers to intervention use	Time constraints/competing life demands (e.g., work; *n* = 4), inability to use the headset or focus due to “really bad” or “intense” sickle cell pain (*n* = 3), distractions in home environment (difficult to use with family or children present; *n* = 3), and being tired, not feeling well, or being hospitalized (*n* = 1)	Inability to use or concentrate on the program due to “high” sickle cell pain or crisis episodes (*n* = 7), time constraints/life demands (*n* = 6), forgetting to use the intervention (*n* = 5), noise in the home environment (from children, family, or household activity; *n* = 3), and being tired (*n* = 1)
Challenges while using the intervention	Technical glitches (e.g., module freezing or not loading; *n* = 2), neck “uneasiness” during one VR session (*n* = 1), and automatic shutdown of the VR device at 20% power level (*n* = 1)	Modules not loading (*n* = 2), had to use the SoundCloud browser because did not have the app (*n* = 1), and a one-time malfunction in one of the SoundCloud links (*n* = 1)
Positive aspects of the intervention	Helped with managing pain (*n* = 5), contained pleasant and immersive visual content (*n* = 5), helped with relaxation and falling asleep (*n* = 4), was calming (*n* = 3) and soothing (*n* = 1), helped to better understand pain and its effects on the body (*n* = 1), and helped with attention and focus (*n* = 1)	Was relaxing and calming (*n* = 12), helped with managing pain (*n* = 10), was easy to use and time convenient (*n* = 10), contained enjoyable narration and relaxation music (*n* = 7), and was accessible (e.g., able to “listen to it anywhere” and able to repeat modules; *n* = 3)
Negative aspects of the intervention	Headset discomfort (e.g., headset feeling heavy or straps feeling tight around back of head; *n* = 4), repetition of content (*n* = 3), and needing a brief period to acclimate to some VR scenes (e.g., underwater, or deep-sea environments; *n* = 2)	Repetition of content (*n* = 7) and mismatch of narrator’s voice (e.g., tone and pace) or background music/sounds (e.g., tempo and style) with user preferences (*n* = 6)

## Data Availability

Data generated and analyzed during this exploratory pilot trial are reported within the manuscript and were made publicly available at https://www.clinicaltrials.gov/ according to the required timelines. Additional data from the study may be obtained from the corresponding author upon reasonable request.

## Supplementary Materials

The following supporting information can be downloaded at: https://www.mdpi.com/article/10.3390/biomedicines14061334/s1, Table S1: Profile of participants’ chronic pain and chronic pain correlates; Table S2: Example interview questions and quotes.

## Abbreviations

The following abbreviations are used in this manuscript:

ADLs	Activities of daily living
ASCQ-Me	Adult Sickle Cell Quality of Life Measurement Information System
AVR	AppliedVR, Inc.
BPS	Biopsychosocial
BRIEF-A	Behavior Rating Inventory of Executive Function—Adult Version
CI	Confidence interval
CONSORT	Consolidated Standards of Reporting Trials
COREQ	Consolidated Criteria for Reporting Qualitative Research
EHRs	Electronic health records
GzMLM	Generalized multi-level linear model
Hb S/β0-Thal	Hemoglobin S Beta 0 Thalassemia
Hb SC	Hemoglobin SC
Hb SS	Hemoglobin SS
IBM	International Business Machines Corporation
MLM	Multi-level linear model
N	Number
PhD	Doctor of Philosophy
PI	Principal investigator
PROMIS	Patient-Reported Outcomes Measurement Information System
QoL	Quality of life
REDCap	Research Electronic Data Capture
SAS	Statistical Analysis System
SCD	Sickle cell disease
SD	Standard deviation
SPSS	Statistical Package for the Social Sciences
U.S.	United States
USA	United States of America
VOC	Vaso-occlusive
VR	Virtual reality
VRSQs	Virtual Reality Sickness Questionnaires
