# Supplementary material for: Preliminary Benefits of In-Home Virtual Reality for Chronic Pain in Sickle Cell Disease: Pilot Randomized Trial
Source: Biomedicines. 2026 Jun 12;14(6):1334. doi: 10.3390/biomedicines14061334 (PMC13296727; doi:10.3390/biomedicines14061334)
Supplement: Supplementary file 1 [file biomedicines-14-01334-s001.zip › Preliminary Benefits of VR in SCD_Table S1_6-10-2026.pdf]

Table S1. Profile of participants' chronic pain and chronic pain correlates.

Measure	Group	Timepoint	*N*	Mean	SD	Min	Max	Measure	Group	Timepoint	*N*	Mean	SD	Min	Max
Chronic Pain Intensity	Virtual reality	Baseline	19	66.84	12.643	46.67	90.00	Chronic Pain Self-Efficacy	Virtual reality	Baseline	19	154.68	27.705	98.00	194.00
		Week 4	12	60.28	13.062	46.67	96.67			Week 4	11	155.82	42.355	70.00	213.00
		Week 8	10	56.33	13.649	40.00	76.67			Week 8	10	166.40	31.010	98.00	205.00
		Week 12	10	60.00	14.055	30.00	76.67			Week 12	10	162.60	24.932	109.00	190.00
	Audio	Baseline	25	63.07	15.838	33.33	100.00		Audio	Baseline	25	155.92	32.597	97.00	220.00
		Week 4	23	54.06	16.236	26.67	93.33			Week 4	23	151.52	35.252	87.00	210.00
		Week 8	23	48.84	17.684	20.00	83.33			Week 8	23	148.13	42.820	75.00	220.00
		Week 12	19	45.96	20.535	3.33	76.67			Week 12	19	160.89	39.329	91.00	220.00
	MLM Effect Tests Group p = 0.029; Time p = 0.003; Group-by-Time p = 0.455								*MLM Effect Tests Group p = 0.636; Time p = 0.762; Group-by-Time p = 0.663*						
Chronic Pain Disability	Virtual reality	Baseline	19	56.14	26.417	13.33	100.00	Social Support	Virtual reality	Baseline	19	111.95	23.813	58.00	149.00
		Week 4	12	48.06	21.671	26.67	100.00			Week 4	11	116.55	27.263	72.00	155.00
		Week 8	10	39.33	17.902	13.33	63.33			Week 8	10	115.50	33.371	44.00	147.00
		Week 12	10	43.00	14.610	23.33	70.00			Week 12	10	121.90	19.941	86.00	151.00
	Audio	Baseline	25	54.67	24.286	0.00	93.33		Audio	Baseline	25	123.48	24.575	64.00	160.00
		Week 4	23	51.01	20.681	16.67	90.00			Week 4	23	129.48	18.783	101.00	160.00
		Week 8	23	44.20	17.062	16.67	76.67			Week 8	23	121.52	22.779	80.00	160.00
		Week 12	29	42.81	27.448	0.00	100.00			Week 12	19	131.89	23.021	72.00	160.00
	MLM Effect Tests Group p = 0.734; Time p = 0.061; Group-by-Time p = 0.930								*MLM Effect Tests Group p = 0.046; Time p = 0.289; Group-by-Time p = 0.888*						
Chronic Pain Grade ≥ 3 (*N*, % reported)	Virtual reality	Baseline	19	57.9%				Health Literacy	Virtual reality	Baseline	19	19.05	1.393	16.00	20.00
		Week 4	12	33.3%						Week 4	11	19.00	1.789	15.00	20.00
		Week 8	10	30.0%						Week 8	10	18.60	2.366	13.00	20.00
		Week 12	10	40.0%						Week 12	10	19.00	1.886	15.00	20.00
	Audio	Baseline	25	72.0%					Audio	Baseline	25	18.24	2.332	12.00	20.00
		Week 4	23	60.9%						Week 4	23	17.65	3.009	9.00	20.00
		Week 8	23	39.1%						Week 8	23	17.04	3.007	11.00	20.00
		Week 12	19	52.6%						Week 12	19	17.84	2.986	10.00	20.00
	[1]*GzMLM Effect Tests Group p = 0.320; Time p = 0.125; Group-by-Time p = 0.795*								*MLM Effect Tests Group p = 0.061; Time p = 0.244; Group-by-Time p = 0.799*						
Pain Catastrophizing	Virtual reality	Baseline	19	22.74	14.802	3.00	52.00	Executive Function	Virtual reality	Baseline	11	46.45	9.191	37.00	71.00

		Week 4	11	18.82	8.195	3.00	30.00			Week 4	6	46.17	11.974	35.00	66.00
		Week 8	10	14.30	7.103	2.00	25.00			Week 8	5	49.20	8.955	37.00	59.00
		Week 12	10	14.50	5.798	5.00	26.00			Week 12	6	42.83	6.274	35.00	49.00
	Audio	Baseline	25	25.48	10.782	2.00	50.00		Audio	Baseline	21	51.62	10.984	36.00	72.00
		Week 4	23	21.91	11.805	0.00	43.00			Week 4	17	46.41	8.163	35.00	63.00
		Week 8	23	19.26	9.631	0.00	33.00			Week 8	17	51.18	9.920	35.00	66.00
		Week 12	19	18.00	11.348	0.00	34.00			Week 12	12	47.50	9.269	37.00	65.00
	MLM Effect Tests Group p = 0.336; Time p = 0.002; Group-by-Time p = 0.983								*MLM Effect Tests Group p = 0.417; Time p = 0.091; Group-by-Time p = 0.267*						
Chronic Pain Acceptance	Virtual reality	Baseline	19	24.00	7.832	13.00	44.00	Depression	Virtual reality	Baseline	19	48.96	8.665	38.20	67.70
		Week 4	11	26.45	6.991	15.00	40.00			Week 4	11	48.59	7.778	38.20	60.30
		Week 8	10	28.20	5.903	21.00	40.00			Week 8	10	45.99	9.708	38.20	63.90
		Week 12	10	28.30	5.012	20.00	38.00			Week 12	10	45.05	8.714	38.20	61.20
	Audio	Baseline	25	21.40	5.972	5.00	32.00		Audio	Baseline	25	55.60	10.033	38.20	75.00
		Week 4	23	23.43	4.804	9.00	32.00			Week 4	23	53.50	9.970	38.20	67.70
		Week 8	23	23.65	6.726	2.00	35.00			Week 8	23	52.94	8.810	38.20	65.80
		Week 12	19	25.26	3.445	17.00	31.00			Week 12	19	49.15	9.557	38.20	64.90
	MLM Effect Tests Group p = 0.160; Time p = 0.010; Group-by-Time p = 0.980								[2]*MLM Effect Tests Group p = 0.268; Time p = 0.049; Group-by-Time p = 0.668*						

**Measure**	**Group**	**Timepoint**	**N**	**Mean**	**SD**	**Min**	**Max**	**Measure**	**Group**	**Timepoint**	**N**	**Mean**	**SD**	**Min**	**Max**
Anxiety	Virtual reality	Baseline	19	53.19	9.328	37.10	70.80	Pain Impact	Virtual reality	Baseline	19	49.65	6.395	38.30	63.80
		Week 4	11	52.65	10.213	37.10	67.70			Week 4	11	51.77	7.071	43.00	63.80
		Week 8	10	47.72	8.449	37.10	59.40			Week 8	10	51.54	6.861	44.40	63.80
		Week 12	10	50.03	11.142	37.10	67.70			Week 12	10	52.59	8.624	36.70	63.80
	Audio	Baseline	25	57.38	8.426	37.10	68.70		Audio	Baseline	25	47.81	6.684	36.70	63.80
		Week 4	23	56.74	9.702	37.10	70.80			Week 4	23	47.30	6.659	34.90	63.80
		Week 8	23	54.65	8.852	37.10	63.50			Week 8	23	48.06	8.427	36.70	63.80
		Week 12	19	52.56	10.834	37.10	66.60			Week 12	19	44.44	9.778	31.00	63.80
	MLM Effect Tests Group p = 0.148; Time p = 0.071; Group-by-Time p = 0.762								*MLM Effect Tests Group p = 0.101; Time p = 0.556; Group-by-Time p = 0.884*						

Sleep Impact	Virtual reality	Baseline	19	49.03	7.043	39.50	61.90	Emotional Impact	Virtual reality	Baseline	19	51.70	7.796	38.50	65.60
		Week 4	11	49.75	7.562	37.30	59.90			Week 4	11	54.56	8.539	38.50	65.60
		Week 8	10	52.91	5.680	45.00	61.90			Week 8	10	55.13	8.658	43.70	65.60
		Week 12	10	55.37	6.148	45.00	61.90			Week 12	10	54.06	6.921	44.90	65.60
	Audio	Baseline	25	49.92	5.485	37.30	58.20		Audio	Baseline	25	49.21	6.447	38.50	65.60
		Week 4	23	50.65	6.203	27.90	61.90			Week 4	23	52.57	8.565	38.50	65.60
		Week 8	23	51.64	5.906	41.40	64.40			Week 8	23	51.53	8.983	39.90	65.60
		Week 12	19	51.88	7.165	35.10	64.40			Week 12	19	52.84	8.656	42.50	65.60
	MLM Effect Tests Group p = 0.812; Time p = 0.005; Group-by-Time p = 0.257								*MLM Effect Tests Group p = 0.441; Time p = 0.237; Group-by-Time p = 0.903*						
Social Functioning	Virtual reality	Baseline	19	49.48	10.516	26.00	69.80	Stiffness Impact	Virtual reality	Baseline	19	47.73	6.618	36.90	59.90
		Week 4	11	56.04	4.562	47.20	62.10			Week 4	11	52.20	7.210	41.30	65.40
		Week 8	10	53.50	8.336	40.40	64.90			Week 8	10	52.02	7.762	41.30	65.40
		Week 12	10	57.82	5.967	50.50	69.80			Week 12	10	54.78	9.234	38.40	65.40
	Audio	Baseline	25	51.43	8.110	36.80	69.80		Audio	Baseline	25	45.78	7.633	35.30	65.40
		Week 4	23	50.33	8.075	36.80	69.80			Week 4	23	46.58	6.129	38.40	65.40
		Week 8	23	51.92	8.938	36.80	69.80			Week 8	23	47.06	6.938	36.90	65.40
		Week 12	19	53.97	10.582	42.10	69.80			Week 12	19	50.48	7.565	39.90	65.40
	MLM Effect Tests Group p = 0.738; Time p = 0.097; Group-by-Time p = 0.229								*MLM Effect Tests Group p = 0.125; Time p = 0.005; Group-by-Time p = 0.817*						
Pain Episode Frequency	Virtual reality	Baseline	19	51.85	8.40	32.41	63.51	Pain Episode Severity	Virtual reality	Baseline	11	49.32	10.36	31.24	61.65
		Week 4	11	50.08	8.22	40.19	63.51			Week 4	10	50.43	9.01	35.92	61.65
		Week 8	10	48.74	10.81	28.53	63.51			Week 8	10	48.55	6.06	40.60	61.65
		Week 12	10	47.96	9.70	32.41	63.51			Week 12	25	45.84	8.00	31.24	59.31
	Audio	Baseline	25	47.65	8.97	28.53	59.62		Audio	Baseline	23	46.19	9.72	31.24	61.65
		Week 4	23	46.27	11.88	28.53	63.51			Week 4	23	44.87	10.94	14.87	59.31
		Week 8	23	45.93	12.83	20.75	63.51			Week 8	19	44.54	8.51	31.24	61.65
		Week 12	19	42.23	12.58	24.64	63.51			Week 12	25	47.65	8.97	28.53	59.62
	MLM Effect Tests Group p = 0.084; Time p = 0.325; Group-by-Time p = 0.914								[2]*MLM Effect Tests Group p = 0.308; Time p = 0.788; Group-by-Time p = 0.578*						

SD: Standard deviation. Min: Minimum. Max: Maximum

[1]Generalized multilevel linear models (GzMLM) performed for binary outcome for Chronic Pain Grade ≥ 3

[2]MLM model effects after adjusting for baseline differences between groups
