# Supplementary material for: Preliminary Benefits of In-Home Virtual Reality for Chronic Pain in Sickle Cell Disease: Pilot Randomized Trial
Source: Biomedicines. 2026 Jun 12;14(6):1334. doi: 10.3390/biomedicines14061334 (PMC13296727; doi:10.3390/biomedicines14061334)
Supplement: Supplementary file 1 [file biomedicines-14-01334-s001.zip › Preliminary Benefits of VR in SCD_Table S2_6-10-2026.pdf]

Table S2. Example interview questions and quotes.

Topics	Questions	VR Group Quotes	Audio Group Quotes
	1. How did the pain program fit in with your daily activities?	"So I tried to do it, um, like nighttime. You know, like before going to bed or once I get home and I get comfortable. Um, you know, before bed where I can relax and just really, you know, there's no really like distractions around. I could just sit-sit back, relax, and just, you know." (30-year-old male with Hb SS)	"…it was very time convenient. It was sent at a particular time, and it was not as necessary for me to use it at that particular time it was sent. So I would either listen to it maybe early in the morning, or maybe before going to bed." (25-year-old female with Hb SS)
Experience of using the intervention	1. What do you think about the pain program? a. What things did you like about it? /What are the most positive aspects of the program?	"So, a couple of things. The convenience of, you know, being at home and being able to use it every day or whenever I'm feeling good enough to use it. Um, so convenience is definitely a plus. Um, I really liked how, you know, the beginning and throughout, you know, it kind of teaches you more about pain, right? 'Cause for the most part, some of us just, you know, oh it's pain, and it's just there, but it really helps you to understand pain more, you know, where it comes, how it's-- how pain affects your body type thing." (30-year-old male with Hb SS)	"Um, what I liked about the program was that it had these, the audios, it was more of like, um, meditation audio that I got to listen to. And they were like short. And yeah, like it was really a fun experience to do that. 'Cause I'd never really thought about like meditating and stuff and that meditating can help me with chronic pain, but it actually did. And um, I think it was also easy for me to use, like, to navigate through and it didn't like take so much of my time. Most of them were like five-- to-- between five to seven minutes long, so it was time-convenient. I could listen to it anywhere and it really helped me a lot. And I think, yeah, it was- it was a wonderful experience for me. Thank you." (22-year-old female with Hb SC)
	b. What things did you dislike about it? /What are the most negative aspects of the program?	"[laughs] Um, it was a-a thing I had to learn, but the-- it was just the voice. It was the way they were talking to me. Like, um, and this is just a personal, like, just my opinion. But like, the voice used kind of like, this is for sure melting your pain, um, or-or helping to control or distract from your pain. And I understand that there may be science behind it, and that's cool, and it's going to be awesome to see that roll out over the years opposed to like. What I grew up with was, you know, just opioids to handle it. Um, but coming at me as someone who has dealt with it my whole life, and then to have this voice	"Um, I guess it was- it was a good thing that, um, it kind of made me more conscious of how, uh, uh, more conscious of my bodily sensations- If that makes sense. Um, it's a good thing, but it was also a bad thing 'cause it made me aware of my pain. [laughs]…my usual, um, distraction method- -which I use heavily, is not thinking about the pain, it's thinking of other things. Um, so I guess that was a plus and a minus. It was good that I became more aware of certain things, or paying more attention to what my body is feeling--but also, I was more aware of

		that's kind of like, this-this is what it is, this is what it's going to be, felt a little annoying at first." (36-year-old female with Hb SS)	what my body was feeling. [chuckles] Does that make sense?" (42-year-old female with Hb SS)
Barriers to intervention use	1. Were there any barriers to use? If so, what were they?	"Well, yeah, one time I was, uh, couple-a times, I was feeling bad. One time I was in the hospital. Uh, for a couple-a days. Lotta---lotta times I just be tired. Like, I'll come home, try to take me a little, small nap, but at the end, you know, by the time I wake up I gotta get ready to go to work or whatever." (44-year-old male with Hb SC)	"Yes. Um, sometimes, uh, I was in pain--from my sickle cell, not from the chronic pain- -and that prevented me from, uh, you know, um, using the-the-the program like when I wanted to, at least I had to wait for my pain to go down or whatever because, you know, um, it gets hard, you know, that sickle cell ain't no joke, so that's about it, honestly, there's no other barriers besides that though." (30-year-old male with Hb SC)
Challenges while using the intervention	1. Did you encounter any issues or challenges while using it?	"…uh, I think maybe once or maybe twice, I had like, you know, some pain--well, not like pain, pain, but had some discomfort in my eyes, and it's probably 'cause I was, you know, using it too much. Or it could be because I was having other things going on at the same time. Um, but I would say that would be the only thing. Everything else was positive." (30-year-old male with Hb SS)	"Um, no, I don't have a SoundCloud app, so I was doing it from a web browser. That would be the only thing, um, since I primarily use Apple Music, I couldn't-- it would be nice to find a way to streamline it to whatever-- like, listening system folks use." (42-year-old female with Hb SC)

[1]Sickle cell disease type: Hb SS: Hemoglobin SS. Hb SC: Hemoglobin SC
